# The potent human CAR activator CITCO is a non-genotoxic hepatic tumour-promoting agent in humanised constitutive androstane receptor mice but not in wild-type animals

**DOI:** 10.1007/s00204-025-03982-9

**Published:** 2025-03-05

**Authors:** C. J. Henderson, A. W. McLaren, A. K. MacLeod, D. Lin, A. R. Cameron, L. R. Chatham, J. Moggs, F. Inesta-Vaquera, John P. Thomson, Richard R. Meehan, P. Chakravarty, M. Schwarz, C. Roland Wolf

**Affiliations:** 1 Division of Systems Medicine, Jacqui Wood Cancer Centre, School of Medicine, University of Dundee, Ninewells Hospital, Dundee, DD1 9SY UK; 2 Drug Discovery Unit, School of Life Sciences, University of Dundee, Dow Street, Dundee, DD1 5EH UK; 3 School of Life Sciences, University of Dundee, Dow Street, Dundee, DD1 5EH UK; 4 Charles River, High Peak Business Park, Buxton Road, Chinley, SK23 6FJ UK; 5 Novartis BioMedical Research, Preclinical Safety, Translational Medicine, Basel, Switzerland; 6 Nicola Murray Centre for Ovarian Cancer Research, Cancer Research UK Scotland Centre, Institute of Genetics and Cancer, University of Edinburgh, Edinburgh, EH4 1QY UK; 7 MRC Human Genetics Unit, Institute of Genetics and Cancer, University of Edinburgh, Western General Hospital, Crewe Rd S, Edinburgh, EH4 1QY UK; 8 The Francis Crick Institute, 1 Midland Road, London, NW1 1AT UK; 9 Department of Experimental and Clinical Pharmacology and Pharmacogenomics, University of Tübingen, Wilhelmstraße 56, 72074 Tübingen, Germany; 10 Innovative Medicines Initiative MARCAR Consortium, Brussels, Belgium

**Keywords:** CITCO, Humanized mice, Non-genotoxic hepatocarcinogen, Constitutive androgen receptor, CAR, Hyperplasia, Rodent carcinogenicity tests

## Abstract

A large number of drugs and compounds produced by the chemical and agrochemical industry, often referred to as ‘non-genotoxic carcinogens’ (NGC), score as tumour promotors in rodent models. It is unclear whether these compounds act similarly in humans. The most extensively investigated compounds have been the anti-convulsive drugs, phenobarbital (PB), and phenytoin. Liver tumours induced by PB are dependent upon the activation of the constitutive androstane receptor (CAR). However, marked species differences in CAR activation by exogenous chemicals exist with some being much more potent activators of human CAR, e.g., 6-(4-chlorophenyl)imidazo[2,1-β][1,3]thiazole-5-carbaldehyde-O-(3,4-dichlorobenzyl)oxime (CITCO). We have compared CITCO-induced tumour formation in the livers of mice in which murine CAR has been replaced with its human counterpart. Our findings reveal that CITCO-dependent liver tumours are only formed in mice-expressing human CAR and not in wild-type animals. In addition, contrary to one of the proposed mechanisms of NGC carcinogenicity, we show that CITCO did not induce a hyperplastic response in the liver of the humanised mice. These data raise some key questions about the mechanism of action of NGCs and identify the limitations of current rodent carcinogenicity test systems in relation to risk assessment.

**Supplementary Information:**

The online version contains supplementary material available at 10.1007/s00204-025-03982-9.

## Introduction

Human carcinogens can exert their carcinogenic activity primarily through a mutagenic “genotoxic” mechanism or, alternatively, through a tumour-promoting “non-genotoxic” mechanism (Luijten et al. 2020). Whilst some carcinogens operating through the former route may also contribute to disease in a non-genotoxic manner, the latter—tumour promotors, but often referred to as non-genotoxic carcinogens (NGC)—are not inherently genotoxic and thus not picked up in short-term genotoxicity assays (Jacobs et al. 2020). A large number of drugs and compounds produced by the chemical and agrochemical industry have been evaluated as non-genotoxic liver carcinogens in rodents and this has resulted in a vigorous debate as to whether these compounds are also tumour promotors in humans (Audebert et al. 2023; Lake 2018; Veltman et al. 2023). Epidemiological studies on epileptics who received prolonged treatment with anti-convulsive drugs phenobarbital (PB) and phenytoin have not demonstrated an increase in liver tumour incidence in these patients [for a review see (La Vecchia and Negri 2014) and references therein]. However, these data are limited and the possibility of a carcinogenic activity of these compounds in humans cannot be ruled out. Induction of liver tumours in rodents by PB is a consequence of the activation of a nuclear receptor, the constitutive androstane receptor (CAR) which then activates the expression of genes which promote carcinogenesis (Braeuning and Schwarz 2020; Cai et al. 2021; Yoshinari and Shizu 2022). There are marked species differences in CAR activation by exogenous compounds, with some being more potent murine Car activators, e.g., 1,4-bis-[2-(3,5-dichloropyridyloxy)] benzene (TCPOBOP) and other more potent activators of the human transcription factor, e.g., 6-(4-chlorophenyl)imidazo[2,1-β][1,3]thiazole-5-carbaldehyde-O-(3,4-dichlorobenzyl)oxime (CITCO) (Honkakoski 2022). Whilst CITCO was identified and subsequently used as a selective human CAR (hCAR) activator (Auerbach et al. 2005), more recent work (Lin et al. 2020) proposes that CITCO can also bind and activate another human hepatic nuclear receptor, PXR (hPXR), albeit in vitro principally using HepG2 and HepaRG cells (Stanley and Wolf 2022). Such species differences raise the possibility that certain compounds which are negative in rodent carcinogenicity studies may be tumour promotors in humans. In this paper, we show that CITCO promoted tumour formation in the livers of mice that are humanised for both CAR and PXR, but not in their wild-type counterparts. These data raise key questions, not only about possible limitations of current rodent test systems, but also about the possible risks associated with human exposure to CAR activators and potentially other tumour promoting agents.

## Materials and methods

Unless otherwise stated, all chemicals were purchased from Sigma-Aldrich (Poole Dorset, UK) and were of the highest quality obtainable. CITCO was purchased from Tocris Bioscience (Abingdon, Oxon, UK), catalogue #3683, purity > 98%. Gurr histological fixative (4% formaldehyde in formal saline) was purchased from VWR Chemicals (Lutterworth, Leics, UK).

### Animals

Mice deleted for Car or Pxr, and/or humanised for CAR and PXR, were generated and characterised as previously described on a C57BL/6NTac background (Ross et al. 2010; Scheer et al. 2010). Cytochrome P450 (CYP) 2B6-LacZ on a C57BL/6J background were maintained as previously described (McMahon et al. 2019).

Mice were kept in open-top cages with a sawdust base, shredded paper and sizzle nest bedding (Datesand, Manchester, UK), ad libitum access to food (RM1; Special Diet Services Ltd., Essex, UK) and drinking water, and acclimatised for at least 5 days before study commencement. Room temperature was between 19 and 23 °C and relative humidity 40–70%, with a 12-h light/dark cycle. Environmental enrichment was provided in the form of nestlets, chew sticks, red plastic tubes/houses, and forage diet (sunflower seeds). In all studies, mice were sacrificed by a rising concentration of CO_2_, and death was confirmed by exsanguination.

Animals were randomly assigned to control or treatment groups; analysts were not blinded to the identity of biological samples. Numbers of animal used were determined using power calculations (G*Power v3.1.9.2; www.gpower.hhu.de) and previous experience. Experimental design was undertaken in line the 3Rs principles of replacement, reduction, and refinement (www.nc3rs.org.uk).

For short-term studies, adult male wild-type (WT) mice, mice humanised for CAR and PXR (hCAR/hPXR) or mice nulled for Car XR and humanised for PXR (Car KO/hPXR), *n* = 3, were treated with corn oil (CO), CITCO (10 or 50 mg/kg), or PB (80 mg/kg) intraperitoneal (IP), daily, 4d and sacrificed on day 5 for blood and tissue collection.

For longer-term studies, adult male WT mice or mice humanised for CAR and PXR (hCAR/hPXR), *n* = 3, were treated with 0.1% (w/v) carboxymethylcellulose (CMC) or CITCO (10 mg/kg), IP, daily for 28d and sacrificed the next day for blood and tissue collection.

For tumour studies, we followed a standard protocol (Moennikes et al. 2000). Adult (8–10 weeks) male WT mice or mice humanised for CAR and PXR (hCAR/hPXR), *n* = 15, were treated with a single IP injection of diethylnitrosamine (N-nitrosodiethylamine, DEN; 90 µg/g body wt.) at the start of the study followed 21 days later by CITCO administered in powdered RM1 diet at the equivalent dose of 10 mg/kg. Another group each of WT and hCAR/hPXR mice were maintained on powdered diet alone as controls. Mice were weighed at least weekly throughout and removed from the study only on veterinary advice or if body weight had decreased by more than 20% from the initial starting weight. Mice were culled by a rising concentration of CO_2_ at 37 weeks, blood samples taken for determination of plasma CITCO levels, and blood chemistry analysis. Livers were dissected, weighed, rinsed in ice-cold PBS, examined grossly for evidence of tumours, and photographed. The gall bladder was removed from the median lobe, which was split in two and fixed overnight in 4% (v/v) *para*-formaldehyde (Gurr; VWR, UK). Fixed samples were then transferred to 70% (v/v) ethanol; one sample was kept in ethanol; the other was processed for wax embedding and sectioning/staining. The left lobe was cut in two; one piece was snap-frozen in liquid nitrogen, whilst the other was placed in a piece of aluminium foil on dry-ice to freeze. The latter was used for cryo-sectioning. The caudate, right anterior, and pyramidal lobes were all snap-frozen in liquid nitrogen and stored at − 80 °C until needed for analysis.

### Preparation of microsomes

Microsomal fractions were prepared from mouse tissues as described previously (Henderson et al. 2019). Briefly, after removal and rinsing in ice-cold PBS, fresh liver samples were homogenised in ice-cold SET buffer (0.25 M sucrose, 5 mM EDTA, 20 mM Tris–HCL, pH 7.4; 9 ml buffer/g liver) and centrifuged at 2000 rpm (Sorvall RTH-250,10 min, 4 °C). The supernatant from this step was further centrifuged (12,000 rpm, Sorvall SS-34 rotor, 20 min, 4 °C) and the resulting supernatant centrifuged at high speed (~ 30,000 rpm, Sorvall TFT-45.6, 90 min 4 °C). The resultant microsomal pellets were resuspended in ice-cold SET buffer and stored at −70 °C prior to protein estimation and subsequent analysis.

### Histology and immunostaining

Cryostat sections (10 µm thickness) were used for all stainings. Seven pairs of two serial sections each were prepared with 200 µm between each of the paired sections of which one was stained for glucose-6-phosphatase (G6Pase) activity to identify tumours deficient in this enzyme. The second section was immunohistochemically stained for glutamine synthetase (GS). Histochemical staining for G6Pase activity was performed according to Wachstein and Meisel (1957). Staining for GS was carried out on formaldehyde-fixed slices according to standard methods (Braeuning et al. 2010), using a primary antibody against GS (1:1000; Sigma, Taufkirchen, Germany) together with an appropriate horseradish peroxidase-conjugated secondary antibody (1:100; Dako, Glostrup, Denmark) with 3-amino-9-ethylcarbazole/H_2_O_2_ as substrates. Nuclei were counterstained by hematoxylin.

### Tumour quantification

Liver tumour burden was quantified by determination of the number and size of G6Pase-altered lesions using an Axio Imager light microscope (Imager.M1; Zeiss, Göttingen, Germany) with AxioVision software Rel.4.5 (Zeiss). Number and size of G6Pase-deficient tumour transections were determined in each of the 7 G6Pase-stained sections and total areas of the respective liver sections were measured for reference. Even though we used 20 sections between the individual slices, we often observed transections from one and the same tumour in several or all sections of the series. In these cases, tumours were counted as only one entity. The area fraction of tumours (tumour/normal tissue) was calculated for each section and averaged for each animal. Immunohistochemical staining for GS was assessed on parallel slices and tumours were classified as GS-positive or GS-negative according to their staining behaviour.

### Blood chemistry

Plasma was prepared from whole blood in heparinised tubes by centrifugation; analyses for alanine aminotransferase (ALT), lactate dehydrogenase (LDH), and albumin were carried out at the Clinical Pathology Service Laboratory (Mary Lyon Centre, Harwell, UK).

### Immunoblotting

Hepatic microsomal samples were separated by sodium dodecyl sulphate–polyacrylamide gel electrophoresis (SDS-PAGE), and CYP and cytochrome P450 oxidoreductase (POR) expression determined by immunoblot analysis using primary antibodies as previously described (Forrester et al. 1992), loading 20 µg of microsomal protein per lane. Positive control standards were membrane preparations from bacteria expressing recombinant murine P450s (1 pmol). Protein expression was visualised using Immobilon Western chemiluminescent detection (Millipore) according to the manufacturer’s instructions, and data were collected and processed (contrast/brightness adjusted identically for each across the entire image) using a Fujifilm LAS-3000 mini-CCD system and the device software Version 2.2. Acquired images were saved in the Tagged Image File Format using software MultiGauge (Fuji Film) and transferred to Power Point.

### Proteomic analysis

Sample preparation, data acquisition, and analysis by targeted high-resolution single ion monitoring (tHR/SIM) in vivo stable isotope labelling by amino acids in cell culture (SILAC) using a pathway-enhanced internal standard were carried out as described previously (MacLeod et al. 2015). Briefly, frozen liver tissue from experimental animals was homogenised in SDT lysis buffer (4% SDS, 0.1 M DTT, 100 mM Tris–HCl, pH7.6), heated to 95 °C for 5 min, sonicated, and debris pelleted by centrifugation at 16,000×*g* for 10 min. The supernatant containing protein for analysis was combined 1:1 with an identically prepared internal standard sample from ^13^C_6_-lysine metabolically labelled mice which had been administered a cocktail of inducers for constitutive androstane receptor (PB), pregnane X receptor (PCN; 5-pregnen-3β-ol-20-one-16α-carbonitrile), and aryl hydrocarbon receptor (bNF; β-naphthoflavone). This sample mixture was separated by SDS-PAGE and each lane cut into three bands for in-gel trypsin-mediated protein digestion. Extracted peptides were analysed using a nanoflow liquid chromatograph (Agilent 1200, Agilent, Santa Clara, CA, USA) with an LTQ-Orbitrap XL (Thermo Fisher Scientific, Waltham, MA, USA), interfaced with a Proxeon nanospray source with a stainless-steel emitter (Thermo Fisher Scientific). For calculation of statistical significance, normalised values were log2-transformed and then analysed by unpaired t test, relative to the WT CMC group, with Holm–Šidák correction for multiple comparisons (Alpha = 0.05). The LC–MS/MS method and tHR/SIM data analysis procedure are described in detail in MacLeod et al. (2015).

Principal components analysis, hierarchical clustering heatmaps, and volcano plots were generated using MaxQuant and Perseus as follows. Xcalibur files were processed using MaxQuant (v1.4.1.2) (Cox and Mann 2008) and the integrated Andromeda search engine with the Uniprot mus musculus (taxID: 10,090) reference proteome set (44,455 entries, downloaded 03.12.14). Cysteine carbamidomethylation was set as a fixed modification, with N-terminal acetylation and methionine oxidation as variable modifications. The protein false discovery rate was set to 1%, minimum peptide length was 7 and a maximum of 2 mis-cleavages was allowed. Data were processed in Perseus (v1.5.1.6). For each control/drug comparison, only proteins with ≥ 3 valid values were retained. Individual animal data were normalised to the mean of the control group, log_2_-transformed and missing values imputed from normal distribution.

### CITCO analysis

#### In vitro metabolism

CITCO (1 µM with 1% acetonitrile) was mixed with liver microsome (0.25 mg/mL) from DEN/CITCO-treated WT and hCAR/hPXR mice in 100 mM potassium phosphate buffer (pH 7.4) containing 3.3 mM MgCl_2_. After pre-incubation (5 min, 37 °C), the reactions were initiated by addition of an NADPH regenerating system (final concentration: 1.3 mM NADPH, 4 mM glucose-6-phosphate, and 2 U/mL glucose-6-phosphate dehydrogenase). CITCO depletion was followed over a 30 min period. An aliquot (100 µL) of reaction mixtures at 1, 5, 10, 20, and 30 min was quenched with the same volume of cold acetonitrile containing 400 ng/mL of triazolam as internal standard (IS). The samples were then centrifuged at 3000*g* for 55 min at 4 °C. An aliquot of the supernatant fraction was analysed by liquid chromatography–tandem mass spectrometry. Incubations were carried out in triplicate.

#### In vivo study

For sample collection, 20 μL of whole blood was withdrawn at the indicated time points. Samples were immediately added to a tube containing heparin solution (20 mL and 15 IU/mL) and put into liquid nitrogen then stored at − 80 °C until processing. On the day of analysis, acetonitrile (160 µL containing 200 ng/mL triazolam) was added to thawed samples which were shaken for 15 min, centrifuged for 10 min at 16,000×*g*, and analysed.

### Liquid chromatography–mass spectrometry–multiple reaction monitoring (LC–MS–MRM) analysis

Analysis of in vitro incubation and in vivo blood samples were carried out by ultra-high-performance liquid chromatography/tandem mass spectrometry (UHPLC/MS–MS) using a Waters Acquity UPLC (Micromass, Manchester, UK) and Micromass Quattro Premier mass spectrometer (Micromass, Manchester, UK) with Electrospray detection. The chromatography was performed using a C18 column (Kinetex^®^ 1.7µ 100A; 50 × 2.1 mm, Phenomenex, Macclesfield, UK) at a temperature of 45 °C with mobile phases of 0.1% formic acid (A) and acetonitrile and 0.1% formic acid (B). A gradient at a flow rate of 0.5 mL/min was run over 3 min as follows: 0–0.5 min: 95% A; 0.5–0.75 min: 95–50% A; 0.75–1.50 min: 50–5% A, 1.50–2.20 min 5% A, 2.20–3 min 5%−95% A, then returning to initial conditions for final 0.5 min. The mass spectrometer was operated in multiple reaction monitoring (MRM), and ion transitions monitored were m/z (mass-to-charge ratio) 435.7 to 260.2 for CITCO, 451.7 to 260.2 Hydroxyl CITCO, and 343.2 to 239.2 for triazolam as internal standard.

### Proliferating cellular nuclear antigen (PCNA) staining

Liver organs were dehydrated and embedded in paraffin and subsequently sectioned at 5 mM thickness using a Shandon Finesse 325 microtome. Sections were mounted in microscopy slides and kept at room temperature (RT) until further processing. For PCNA staining (DAKO, M0879), slides were deparaffinized with xylene and re-hydrated through a graded series of 100–50% (v/v) ethanol solutions, followed by 10 min boiling in citrate buffer (10 mM, pH 6) for antigen retrieval. Slides were subsequently permeabilized in Triton buffer (0.3% Triton X-100, 100 mM glycine) for 20 min at RT and washed in PBS before endogenous enzymes blocking (10 min, DAKO Dual Endogenous Enzyme Block buffer) as suggested by the manufacturer, washed with PBS, and incubated in goat serum non-specific antigen blocking buffer for 1 h at RT. The primary antibody (1:200 dilution in 5% bovine serum albumin/PBS buffer) was incubated over night at 4° and washed before polymer-HRP (DAKO) applied for 30 min according to the manufacturer’s instructions. The diaminobenzidine (DAB) + chromogen reaction was followed for 5 min under the microscope and stopped by slide immersion in distilled water and washed for 5 min under running water. Slides were then counterstained using haematoxylin buffer, and dehydrated washed in distilled water, dehydrated through 95% (v/v) ethanol and two changes of 100% (v/v) ethanol, cleared with two changes of xylene, air-dried, and mounted in DPX medium (Sigma).

### ImageJ quantitation

The software ImageJ v1.51q was used for quantitative image analysis of the PCNA staining. The densitometric analysis was based on microscope images which were acquired with a Zeiss Axio Observer (Carl Zeiss, Jena, Germany). For each untreated or treated mouse liver sample, five different fields were acquired. Captures were converted to 8-bit images, background subtracted, signal threshold applied, and converted to mask. Subsequently, the software was automatically determined particles greater than 50 pixels and average number of positive cells and standard deviation was plotted. Average values were compared by a Student’s *t* test.

### RNAseq analysis

RNA was extracted from liver tissue using TRIzol (Thermo Fisher Scientific) and according to the manufacturer’s instructions. RNAseq was carried out at the Wellcome Trust Clinical Research Facility (Edinburgh) and data files analysed at the Francis Crick Institute (London). FASTQC version 11.7 was used to assess FASTQ file quality.

Trimmomatic version 36 was used to trim adapter sequences from Fastq files.

Alignment to Ensembl Mus_musculus.GRCm38.89 was performed using STAR 2.5.2 and RSEM version 1.3.

Raw counts matrix was generated using in house scripts.

All downstream analysis was done using DESeq2 version 1.32 and R version 4.1.0 (2021-05-18). Differential analysis was done using DESEQ2 Wald’s test. Genes were called if the adjusted *p* value was less than 0.05.

## Results

**Fig. 1:**
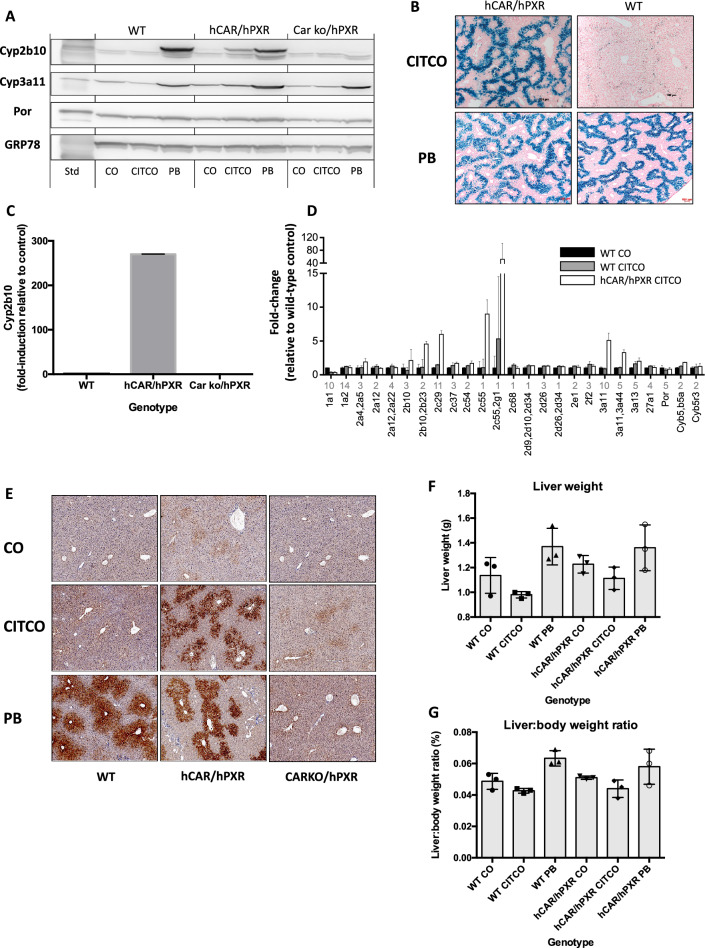
**a** Adult, male wild-type (WT) mice, mice humanised for CAR and PXR (hCAR/hPXR) and mice nulled for Car and humanised for PXR (Car ko/hPXR), n = 3, were treated with corn oil (CO), CITCO (10mg/kg, ip, daily for 4d) or phenobarbital (PB; 80mg/kg, ip daily, 4d) and sacrificed on day 5. Hepatic microsomal samples were pooled and immunoblotted as described in the Materials and Methods for Cyp2b10, Cyp3a11, P450 oxidoreductase (Por), and using GRP78 as loading control. **b** Transgenic mice carrying a CYP2B6-LacZ reporter and either wild-type (mCar/mPxr) or humanised for CAR and PXR (hCAR/hPXR) were treated with CITCO (10mg/kg, ip, daily for 4d) or phenobarbital (PB; 80mg/kg, ip daily, 3d), and sacrificed the following day. Liver tissue was cryosectioned and stained for β-galactosidase activity as described in Materials and methods. **c** Induction of Cyp2b10 RNA expression in adult, male wild-type (WT) mice, mice humanised for CAR and PXR (hCAR/hPXR) and mice nulled for Car and humanised for PXR (Carko/hPXR), n = 3, were treated with CITCO (50mg/kg, ip, daily for 4d) and sacrificed on day 5. **d** Proteomic analysis was carried out as described in the Materials and Methods on liver tissue from adult, male wild-type (WT) or mice humanised for CAR and PXR (hCAR/hPXR) (n = 3), were treated with CO or CITCO (10mg/kg, ip, daily for 4d) and sacrificed on day 5. Data shows fold induction (mean ± SD) of cytochrome P450 proteins, relative to WT vehicle control; numbers under Xaxis for each data set indicate the number of unique SILAC peptides used for quantification of that protein. **e** Adult, male wild-type (WT) mice, mice humanised for CAR and PXR (hCAR/hPXR) and mice nulled for Car and humanised for PXR (Carko/hPXR), n = 3, were treated with CO, CITCO (10mg/kg, ip, daily for 4d) or phenobarbital (PB; 80mg/kg, ip daily, 4d) and sacrificed on day 5. Liver tissue was fixed and processed for Cyp2b10 immunohistochemistry as detailed in Materials and Methods. **f** Liver weights, **g** liver-body weight ratio for mice treated in **e**; n = 3, symbols for each group indicate data points, error bars mean ± SD, and unpaired ttest indicates no significant difference for CITCO- or PB-treated groups relative to appropriate CO-treated control group

To establish the role of the transcription factor CAR in the regulation of gene expression by CITCO, WT and humanised hCAR/hPXR and Car null/hPXR mice animals were treated for 4 days with vehicle, CITCO, or phenobarbital (PB). Based on Western blotting (Fig. 1A), reporter gene expression (Fig. 1B), RNA expression (Fig. 1C), proteomics (Fig. 1D), and immunohistochemistry (Fig. 1E), hepatic genes regulated by CAR, including Cyp2b10 and Cyp3a11, were highly induced by CITCO in hCAR/hPXR mice but at best only very weakly in WT animals. In contrast, PB, used as a positive control, induced the expression of CAR regulated genes in both the WT and hCAR/hPXR mice with equal efficacy and the changes in gene expression were attenuated in Car null/hPXR animals (Fig. 1A, C, E). These findings are consistent with reports in the literature (Braeuning et al. 2014; Braeuning and Pavek 2020; Yamamoto et al. 2004). It is unclear why, but interesting to note, that the population of hepatocytes in which Cyp2b10 expression was induced in hCAR/hPXR mice appeared to differ between PB (pericentral localisation, zone 3) and CITCO (mid-zonal, zone 2) treatment (Fig. 1E).

It has been proposed that the induction of hepatocyte proliferation and associated hyperplasia and liver growth is a key element in the adverse outcome pathway of PB-mediated liver tumour promotion in rodents (Yamada et al. 2015), but the situation in humans is less certain (Braeuning 2014; Braeuning and Schwarz 2016; Elcombe et al. 2014). In terms of liver weight and liver-to-body weight ratio, PB treatment increased both parameters in WT and hCAR/hPXR mice relative to vehicle control, albeit non-significantly, whereas no such change was observed in either WT or hCAR/hPXR animals following CITCO treatment, with both parameters decreasing (non-significantly) relative to vehicle control (Fig. 1F, G). Moreover, there was no increase in Ki67-labelling index in liver of CITCO- (4 days) treated hCAR/hPXR mice in contrast to what was seen in WT and in hCAR/hPXR animals treated with PB (data not shown). Blood chemistry data (alanine aminotransferase (ALT), bilirubin, lactate dehydrogenase (LDH), creatinine, and glucose for this study are shown in Supplementary Fig. 1. Although some of these biochemical parameters showed slight elevations in some cases, none were statistically significant with respect to relevant control treatment.

**Fig. 2:**
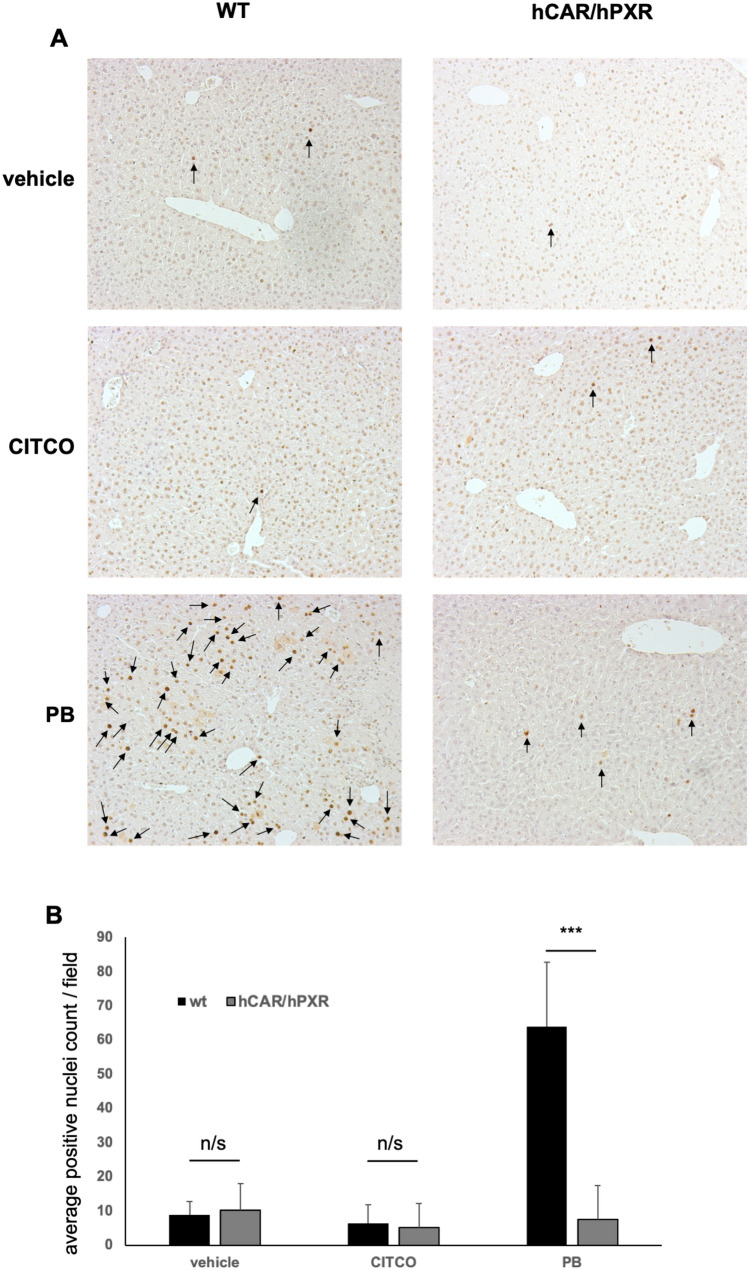
**a** Representative liver PCNA staining images (10x) from duplicate wild type (WT) or hCAR/hPXR mice treated with vehicle, CITCO or phenobarbital (PB) for 4d as described in Figure 1 and Materials and Methods. **b** Quantitation of PCNA positive nuclei from multiple liver sections as shown in (**a**). Data was obtained after processing 5 different fields of PCNA staining images for each mouse and treatment using ImageJ. Black arrows indicate positive stained nuclei; n/s: non-significant; ***, *p* value < 0.001.

To further evaluate whether CITCO provoked cell-cycle effects and a cellular response, liver sections from WT and hCAR/hPXR mice treated with vehicle, CITCO or PB for 4d before sacrifice were processed, fixed and stained for the presence of proliferating cell nuclear antigen (PCNA), and the number of cells with PCNA-positive nuclei evaluated as described in Materials and methods; the results are shown in Fig. 2. The number of PCNA-positive cells was not significantly different between WT and hCAR/hPXR mice treated with vehicle or CITCO. However, with PB treatment, PCNA positivity was significantly elevated in WT compared to hCAR/hPXR mice, with the number of PCNA-positive cells in the latter animals being approximately of the same magnitude as in the other treatment groups (Fig. 2).

Analysis of RNAseq data from WT and hCAR/hPXR mice treated with CITCO for 4d found less than 50 genes whose expression was changed in hCAR/hPXR mice greater than 1.5-fold up or down relative to WT (Supplementary Table 1). Perhaps unsurprisingly, most of these genes (including CAR and PXR themselves) were associated with CAR- or PXR-related pathways (> 10/20 pathways). Consistent with data presented earlier (Fig. 1), the expression of several P450s was elevated—Cyp3a25 (2.5-fold), Cyp2c29 (fourfold), Cyp3a11 (4.6-fold), Cyp3a59 (5.6-fold), and Cyp2b10 (57-fold). Ugt1a1 was found to be strongly induced, the fold-change of more than 4.3 million presumably representing an increase in expression from an almost non-existent baseline; although almost nothing is known about CITCO metabolism, it is interesting to speculate that Ugt1a1 may be involved. Further analysis by gene set enrichment analysis also linked to a number of CAR- and PXR-related pathways but showed no consistent significant changes in cell-cycle-related gene expression (Supplementary Table 2).

**Table 1:** Tumour burden in wild-type (WT) and mice humanised for CAR and PXR (hCAR/hPXR) following treatment with diethylnitrosamine (DEN) ± CITCO as described in “Materials and methods”

Genotype	Treatment	# Mice	# Mice with tumours (%)	Total # tumours/group		Diameter of tumours (mm)		Area fraction of tumours (%)	
				GS^+ve^	GS^−ve^	GS^+ve^	GS^−ve^	GS^+ve^	GS^−ve^
WT	DEN	10	0	0	0	0	0	0	0
WT	DEN + CITCO	8	0	0	0	0	0	0	0
hCAR/hPXR	DEN	13	0	0	0	0	0	0	0
hCAR/hPXR	DEN + CITCO	12	6 (50)	28^a^	10^a^	0.58 ± 0.45^b^	0.90 ± 0.40^b^	3.24 ± 8.4^b^	4.85 ± 5.23^b^

^a^one of the 6 tumour-bearing mice had 21 tumours, of which 18 (86%) were GS^+ve^

^b^Data shown are mean ± SD

**Fig. 3:**
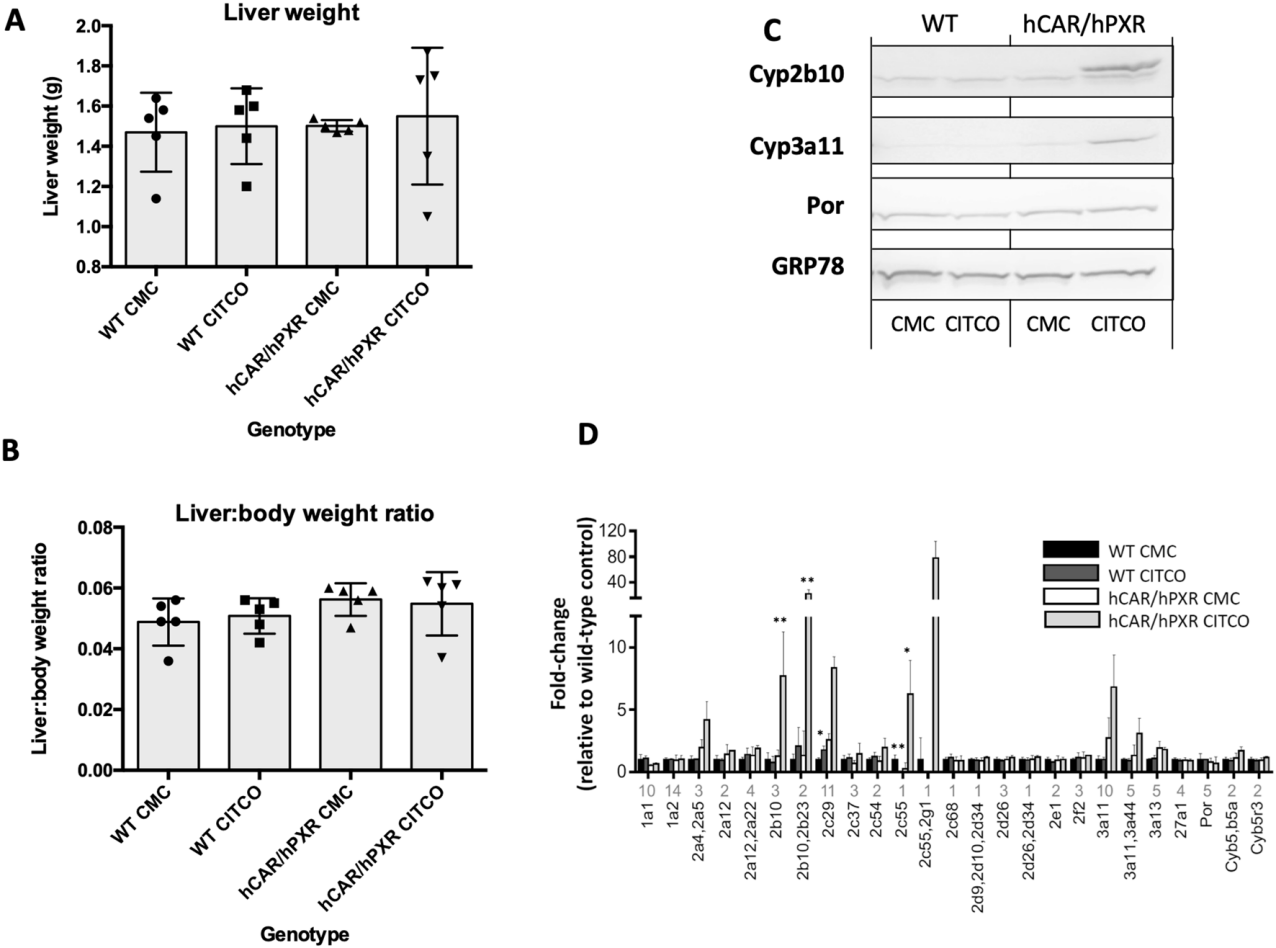
**a** Adult, male wild-type (WT) mice and mice humanised for CAR and PXR (hCAR/hPXR), n = 3, were treated with 0.1% (w/v) carboxymethylcellulose (CMC) or CITCO (10mg/kg, ip, daily for 28d) and sacrificed the next day. **a** Liver weights; **b** liver-body weight ratio. **c** Hepatic microsomal samples were pooled and immunoblotted as described in the Materials and Methods for Cyp2b10, Cyp3a11, P450 oxidoreductase (Por), and using GRP78 as loading control. **d** Proteomic analysis was carried out as described in the Materials and Methods on liver tissue from adult, male wild-type (WT) or mice humanised for CAR and PXR (hCAR/hPXR) (n = 3), treated with CMC or CITCO (10mg/kg, ip, daily for 28d) and sacrificed the next day. Data shows fold induction (mean ± SD) of cytochrome P450 proteins, relative to WT vehicle control; numbers under X-axis for each data set indicate the number of unique SILAC peptides used for quantification of that protein. Analysis by *t*-test, * = *p* < 0.05; ** = *p* < 0.01

We next investigated the effects of CITCO administration over a longer period, dosing WT, and hCAR/hPXR mice for 28d before sacrifice and analysis. The effects of this treatment protocol on liver weights and liver:body weights are shown in Fig. 3A, B, respectively. Whilst the highest liver weights were found in CITCO-treated hCAR/hPXR mice, the range of liver weights meant that there was no significant difference between this group and any of the others in the study (Fig. 3A). Similarly, liver:body weights ratios showed no significant differences with respect to treatment or genotype (Fig. 3B).

Cytochrome P450 expression was then examined in 28d CITCO-treated hCAR/hPXR and WT mice. CITCO treatment strongly induced expression of Cyp2b10 by immunoblotting (Fig. 3C) and proteomic analysis (Fig. 3D), whilst Cyp3a11 was also induced, albeit more modestly. Comparing within a genotype, only two P450s—Cyp2c29 (increase) and Cyp2c55 (decrease)—examined by proteomic analysis were significantly altered in expression relative to control in WT mice following CITCO treatment (t test). Interestingly, a number of P450s were found with elevated expression levels in hCAR/hPXR mice following 28d CITCO treatment (Fig. 3D), including Cyp2a4, Cyp2a5, Cyp2g1, Cyp3a11, Cyp3a44, Cyp2b10, Cyp2b23, Cyp2c29, and Cyp2c55, although only the latter four were statistically significant (*t* test, *p* < 0.05).

Blood chemistry for alanine aminotransferase (ALT), aspartate aminotransferase (AST), bilirubin, and lactate dehydrogenase (LDH) in these animals is shown in Supplementary Fig. 2. None of the biochemical parameters measured showed significant changes with respect to the WT control group.

**Fig. 4:**
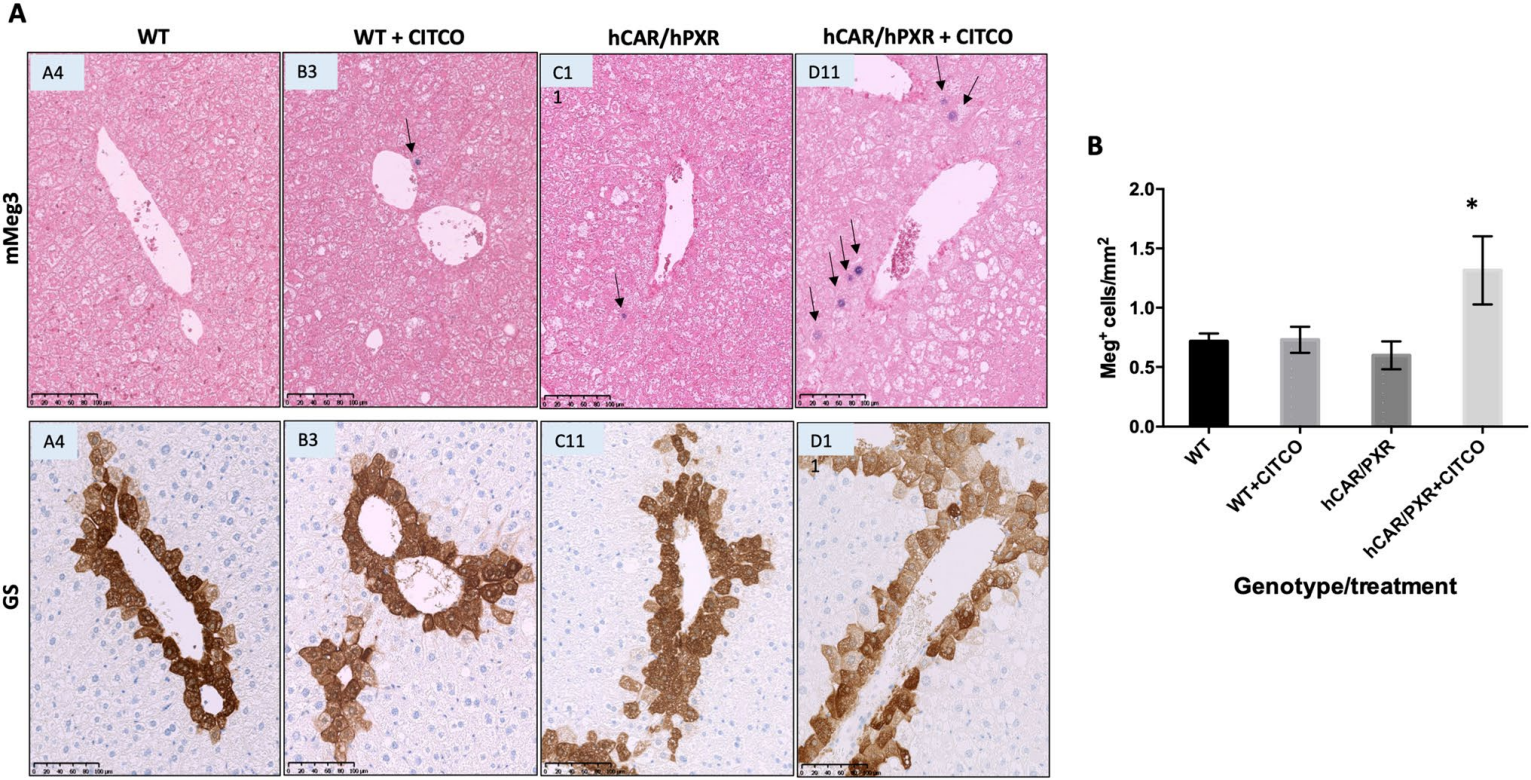
**a** In situ hybridisation for Meg3 (upper panels, arrows) and immunohistochemistry for glutamine synthetase (GS) (lower panels) in liver sections from wild-type (WT) mice and mice humanised for CAR and PXR (hCAR/hPXR) following treatment with diethylnitrosamine (DEN) ± CITCO as described in Materials and Methods. Scale bar = 100μM. **b** Quantification of Meg3 staining; statistical significance determined by unpaired *t*-test

We have previously shown that NGC-induced epigenetic changes, mediated by CAR, provide a signature for NGC activity following exposure to PB, including demethylation at the Meg3/Dlk1 gene locus as well as global changes in DNA methylation profiles (Lempiainen et al. 2013; Luisier et al. 2014; Thomson et al. 2014). Following 28 days of CITCO treatment, an increase in the expression of Meg3 was observed by in situ hybridisation in the hCAR/hPXR mice but not in WT animals (Fig. 4A, arrow) and when quantified (Fig. 4B) found to be significantly higher in hCAR/hPXR mice treated with CITCO compared to those administered only vehicle.

**Fig. 5:**
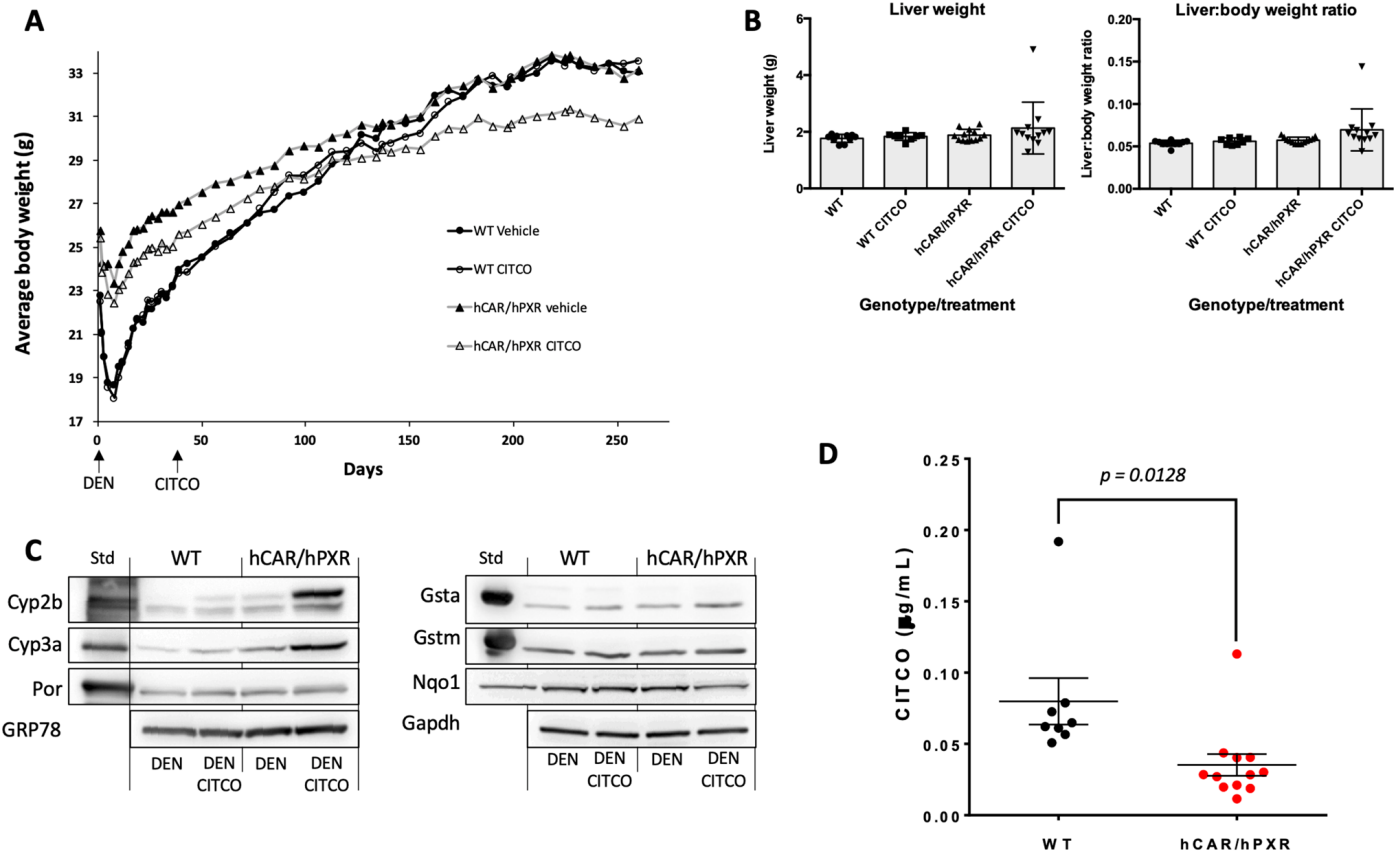
**a** Mean body weight of wild-type (WT) mice and mice humanised for CAR and PXR (hCAR/hPXR) following treatment with diethylnitrosamine (DEN) ± CITCO as described in Materials and Methods. **b** Liver weight and liver:body weight ratios of WT and hCAR/hPXR mice treated with DEN ± CITCO (mean ±SD). Symbols indicate average weight of animals in a group at each timepoint. **c** Immunoblotting of pooled (n = 3) liver microsomes (left) and cytosol (right) fractions for Phase I and Phase II enzymes. **d** Terminal plasma CITCO concentrations in WT and hCAR/hPXR mice. Data from individual animals are indicated by the symbols, mean ± SD is indicated for each group. Statistical significance determined by unpaired *t*-test

We then carried out a carcinogenicity study with CITCO in WT and hCAR/hPXR mice. A standard protocol was used (Moennikes et al. 2000) involving an initial dose of the carcinogen N-nitrosodiethylamine (DEN) followed by 37 weeks of dietary CITCO. In the initial stages of the study, both groups of humanised mice were, on average, heavier than mice in the WT groups (Fig. 5A). However, as the study progressed both vehicle- and CITCO-treated WT mice gained weight and by the termination of the study, the average weights of these groups were indistinguishable from those of vehicle-treated hCAR/hPXR mice, whereas hCAR/hPXR mice treated with CITCO failed to gain as much weight and were on average lighter than their vehicle-treated counterparts (Fig. 5A).

Neither CITCO treatment nor genotype changed average liver weight nor liver:body weight ratios as these were not significantly different between groups (Fig. 5B). Western blotting for Cyp2b proteins showed marginally increased expression of Cyp2b10 in WT mice following CITCO treatment, to approximately the same level as that found in DEN-treated hCAR/hPXR mice, whereas additional treatment with CITCO resulted in significantly increased expression of Cyp2b10 as well as Cyp3a11 (Fig. 5C). For comparison, there was no change in P450 Reductase (Por) levels across the groups. Similarly, Western blots for Gsta, Gstm, and Nqo1 showed similar expression levels regardless of treatment or genotype.

Interestingly, when terminal plasma CITCO was measured, CITCO levels were found to be significantly (*p* < 0.05) lower in hCAR/PXR mice than in the WT group (Fig. 5D). It could be speculated that this is due to CITCO inducing its own metabolism in CAR humanised mice, although little is known about the metabolism of CITCO.

**Fig. 6:**
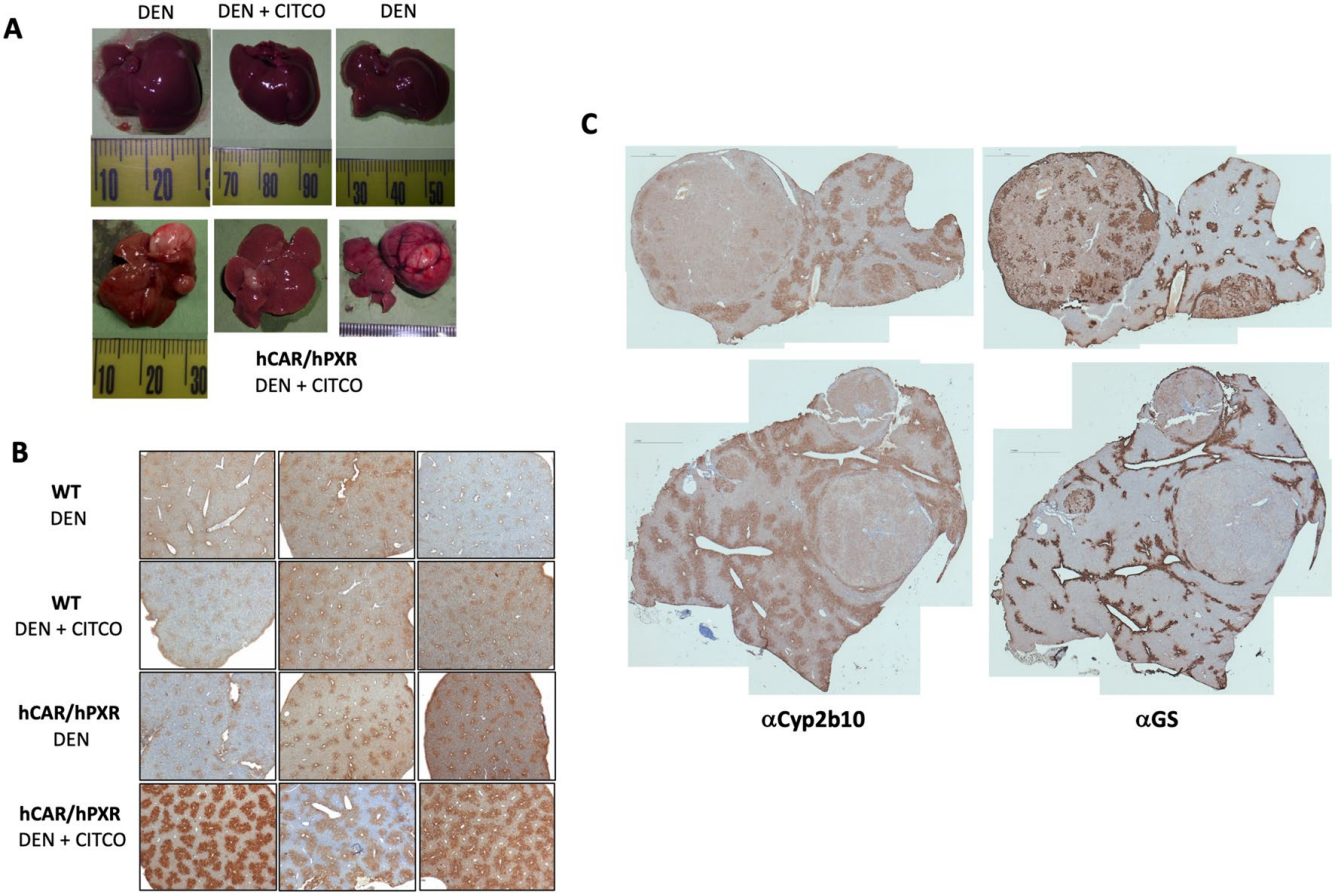
**a** Representative gross liver morphology from wild-type (WT) mice humanised for CAR and PXR (hCAR/hPXR) following treatment with diethylnitrosamine (DEN), and hCAR/hPXR mice treated with DEN + CITCO as described in Materials and Methods (upper panels). Lower panels – liver tumours from individual hCAR/hPXR mice treated with DEN + CITCO. **b** Immunohistochemistry of liver sections from WT and hCAR/hPXR mice treated with DEN ± CITCO for Cyp2b10. Bright field magnification x5, sections shown representative of each group. **c** Composite low-field photomicrograph of immunohistochemical staining for Cyp2b10 or glutamine synthetase (GS) of liver tumour sections from two different hCAR/hPXR mice (upper and lower panels) treated with DEN ± CITCO. Scale bar = 1mM

Following 37 weeks of CITCO exposure, none of the livers from hCAR/hPXR animals treated with DEN alone and WT groups treated with DEN followed by CITCO showed any pathological changes. However, 50% of the CITCO-treated hCAR/PXR animals had liver adenomas with three of the mice exhibiting multiple adenomas and a very high tumour burden, (Table 1; Fig. 6A). In support of the data shown in Fig. 1, areas of histologically normal liver exhibited high levels of Cyp2b10 in DEN/CITCO-treated hCAR/hPXR mice but much lower levels in DEN or DEN/CITCO-treated WT or in hCAR/hPXR mice treated with DEN alone (Fig. 6B).

Phenotypically, the majority of tumours (> 85%, 18/21) were glutamine synthetase-positive, well-differentiated hepatocellular adenoma (Fig. 6C). This tumour phenotype is characteristic of PB-type promoted liver tumours and is a consequence of b-catenin activation resulting from mutations in exon 3 of the *Ctnnb1* gene (Aydinlik et al. 2001; Braeuning et al. 2014; Braeuning and Pavek 2020; Hailfinger et al. 2006).

## Discussion

We have observed that CITCO, as well as in a previous publication PB (Braeuning et al. 2014), promoted the occurrence of liver tumours in mice where the only genetic difference is the substitution of murine CAR and PXR for their human counterparts. These data clearly demonstrate that these human receptors can mediate tumour-promoting activity in mouse liver and that the potency of CAR activation is a critical factor in this process. The observation that a wide range of non-mutagenic compounds induce liver tumours in rodents is the rationale behind the regulatory requirement for 2-year carcinogenicity studies to identify potential carcinogens in humans (Calabrese et al. 2021). The interpretation of such studies in relation to risk assessment is the subject of significant importance and often intense debate (Lake 2018). There is substantial evidence that rodents, and in particular certain strains of mice, such as C3H or B6C3F1 mice, are highly susceptible to NGC-mediated tumour promotion, suggesting that a carcinogenic response seen in these strains cannot be directly translated into humans (Dragani et al. 1995; Drinkwater and Ginsler 1986). However, the hCAR/hPXR mice of the present study were generated in a C57BL6 strain, known to be resistant to hepatic tumour promotion (Bursch et al. 2004; Jones et al. 2009), which more closely reflects the human situation.

We contribute to this debate by showing that the potency of a compound in the activation of the transcription factor CAR is a major determinant in NGC-mediated tumorigenic potential. This suggests that some compounds specific for the human CAR receptor, such as CITCO, will be negative in carcinogenicity studies using WT mice but positive in a humanised CAR model. This also implies that some compounds, which are weak activators of human CAR but strong activators of its murine counterpart, will be carcinogenic in WT mice but negative in a humanised CAR animal and therefore probably of no risk to humans. These findings identify systematic shortcomings in the predictive power of current carcinogenicity tests.

At the present time, the NGC-activated pathways which are essential for tumorigenesis have not been unequivocally identified and a variety of mechanisms have been proposed. These include induction of liver cell proliferation, either receptor-mediated or regenerative following induction of hepatotoxicity, blockage of gap junctional intercellular communication, changes in micro-RNA expression and/or DNA methylation, inhibition of apoptosis, or induction of oxidative stress (Felter et al. 2018; Luijten et al. 2020; Thomson et al. 2014). Major emphasis has been placed on the ability of the compounds to induce replicative DNA synthesis`, which by association`, although not usually measured`, has been extrapolated to imply an increase in mitosis and cell division. A recent proteomic study used PB-treated chimeric FRG-KO mice, a line where deletion of fumarate acetoacetate hydrolase (Fah) causes accumulation of a toxic metabolite and destruction of murine hepatocytes, on a genetic background (Rag2 and Interleukin 2 Receptor Gamma null) which allows repopulation to a large extent with human hepatocytes; the authors reported evidence for hepatocellular proliferation in the remaining ‘murine but not human`, hepatocytes (Sprenger et al. 2022). Although the induction of hepatocyte cell division may be a contributing factor to the tumorigenic effects, it does not seem to be essential as in the current work, and previously reported, NGC-induced tumour formation is observed in the absence of a hyperplastic response in liver (Luijten et al. 2020). In the current work, it is important to note that we found no evidence from cell cycle gene expression of significant hepatocyte proliferation with CITCO treatment, nor did we find increased liver weight or liver:body weight ratios at the conclusion of the carcinogenicity study despite half of the hCAR/hPXR group developing tumours (Fig. 5B). It is also of note that circulating levels of CITCO were significantly lower in the hCAR/hPXR group treated with DEN/CITCO compared to WT mice treated in the same way (Fig. 5E). The reasons for this are unclear, and although it is tempting to speculate that this may be due to increased P450 expression in the hCAR/hPXR group following extended CITCO treatment (Figs. 3, 5E), little is known regarding CITCO metabolism and the lower plasma concentration may be a consequence of other metabolic or physiological process, i.e., renal clearance.

We would argue that without a greater understanding of the mechanism of carcinogenicity, current human-relevant model systems, for example using human cell lines, hepatocytes, or chimeric animals transplanted with human hepatocytes, which measure the capacity of a compound to induce DNA synthesis (which in their own right have a number of significant limitations) do not rule out the possibility that a human CAR activator might act as a non-genotoxic tumour-promoting agent in humans. Whether a compound is of risk to humans must be considered in relation to the level and duration of exposure and whether it has the potential to activate other pathways which may contribute to its tumorigenic potential, either biochemical, pharmacological, or toxicological. Mechanistic studies to date have been almost exclusively carried out on one compound, PB, whose properties other than the capacity to activate CAR will no doubt be different to many other NGCs. Consequently, our understanding of the mechanistic basis of NGC based on studies with PB alone does not justify the conclusion that species differences on the effects of CAR activators in human liver are so fundamentally different to those observed in rodents to conclude that human CAR activators will not be a carcinogenic risk to humans. A number of fundamental questions remain to be answered. For example, why do CAR activators often induce different sub-sets of genes? What is the relationship between CAR activating potency and the activation of these pathways? Does this relate to different CAR conformers based on the mode of activation [direct or indirect (Sato et al. 2022)] or the different structural changes on ligand binding which allows CAR to bind to different enhancer elements in gene promoters? There is much still to be learnt before the currently available in vivo and in vitro assays can be considered predictive of human response. Humanised mouse models provide a powerful approach to increase this understanding.

## Supplementary Information

Below is the link to the electronic supplementary material.

Supplementary file1 (DOCX 5272 KB)

Supplementary file2 (DOCX 5151 KB)

Supplementary file3 (DOCX 21 KB)

Supplementary file4 (DOCX 17 KB)

## Data Availability

Gene expression data generated in thsi study has been submitted to the Gene EXpression Omnibus (GEO), under the code GSE279414.
