# Correction: The potent human CAR activator CITCO is a non-genotoxic hepatic tumour-promoting agent in humanised constitutive androstane receptor mice but not in wild-type animals

**DOI:** 10.1007/s00204-026-04463-3

**Published:** 2026-06-27

**Authors:** C. J. Henderson, A. W. McLaren, A. K. MacLeod, D. Lin, A. R. Cameron, L. R. Chatham, J. Moggs, F. Inesta‑Vaquera, John P. Thomson, Richard R. Meehan, P. Chakravarty, M. Schwarz, C. Roland Wolf

**Affiliations:** 1 Division of Systems Medicine, Jacqui Wood Cancer Centre, School of Medicine, University of Dundee, Ninewells Hospital, Dundee, DD1 9SY UK; 2 Drug Discovery Unit, School of Life Sciences, University of Dundee, Dow Street, Dundee, DD1 5EH UK; 3 School of Life Sciences, University of Dundee, Dow Street, Dundee, DD1 5EH UK; 4 Charles River, High Peak Business Park, Buxton Road, Chinley, SK23 6FJ UK; 5 Novartis BioMedical Research, Preclinical Safety, Translational Medicine, Basel, Switzerland; 6 Nicola Murray Centre for Ovarian Cancer Research, Cancer Research UK Scotland Centre, Institute of Genetics and Cancer, University of Edinburgh, Edinburgh, EH4 1QY UK; 7 MRC Human Genetics Unit, Institute of Genetics and Cancer, University of Edinburgh, Western General Hospital, Crewe Rd S, Edinburgh, EH4 1QY UK; 8 The Francis Crick Institute, 1 Midland Road, London, NW1 1AT UK; 9 Department of Experimental and Clinical Pharmacology and Pharmacogenomics, University of Tübingen, Wilhelmstraße 56, 72074 Tübingen, Germany; 10 Innovative Medicines Initiative MARCAR Consortium, Brussels, Belgium

**Correction to: Archives of Toxicology (2025) 99:2197–2210** **https://doi.org/10.1007/s00204-025-03982-9**

In Figure 1B of this article, the bottom left panel (hCAR/hPXR + PB) was previously published in McMahon et al. (2019) Molecular Pharmacology paper (https://doi.org/10.1124/mol.118.113555) Figure 3A (bottom left panel, hCAR/hPXR + PB)), by the same lab. The image was reused here for illustrative purposes to demonstrate LacZ expression in the CYP2B-LacZ reporter mouse model following phenobarbital treatment. Although the use of this model was described in the Materials and Methods section, referencing McMahon et al. (2019), the prior publication of this specific image was not explicitly acknowledged, and for this we apologise. This clarification does not affect the results or conclusions of the article.

**Fig. 1:**
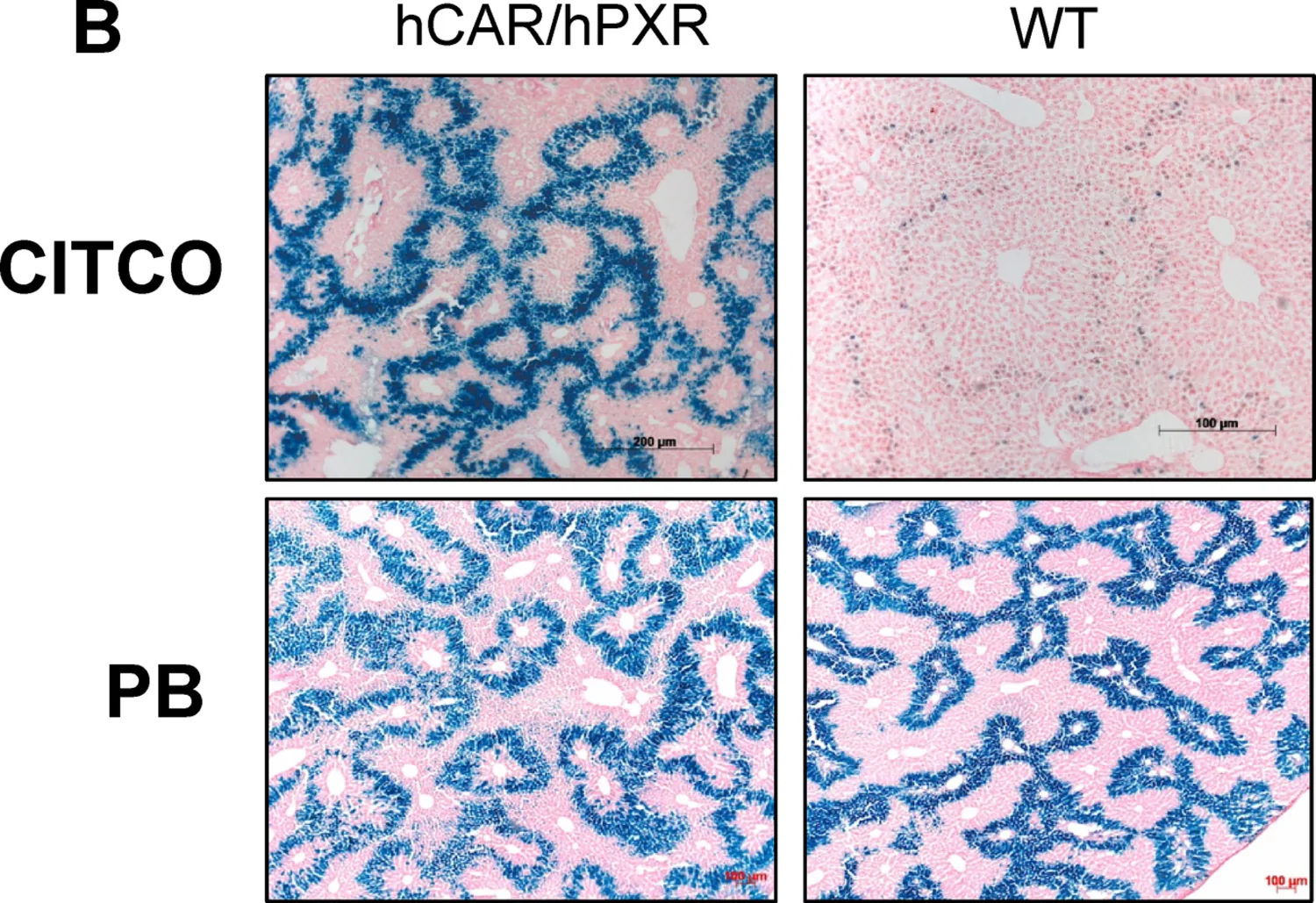
**A**: … **B**: Transgenic mice carrying a CYP2B6-LacZ reporter and either wild-type (mCar/mPxr) or humanised for CAR and PXR (hCAR/hPXR) were treated with CITCO (10 mg/kg, ip, daily for 4d) or phenobarbital (PB; 80 mg/kg, ip daily, 3d), and sacrificed the following day. Liver tissue was cryosectioned and stained for β-galactosidase activity as described in Materials and methods

