# Supplementary material for: The potent human CAR activator CITCO is a non-genotoxic hepatic tumour-promoting agent in humanised constitutive androstane receptor mice but not in wild-type animals
Source: Arch Toxicol. 2025 Mar 5;99(5):2197–210. doi: 10.1007/s00204-025-03982-9 (PMC12085376; doi:10.1007/s00204-025-03982-9)
Supplement: Supplementary file 1 — Supplementary file1 (DOCX 5272 KB) [file 204_2025_3982_MOESM1_ESM.docx]

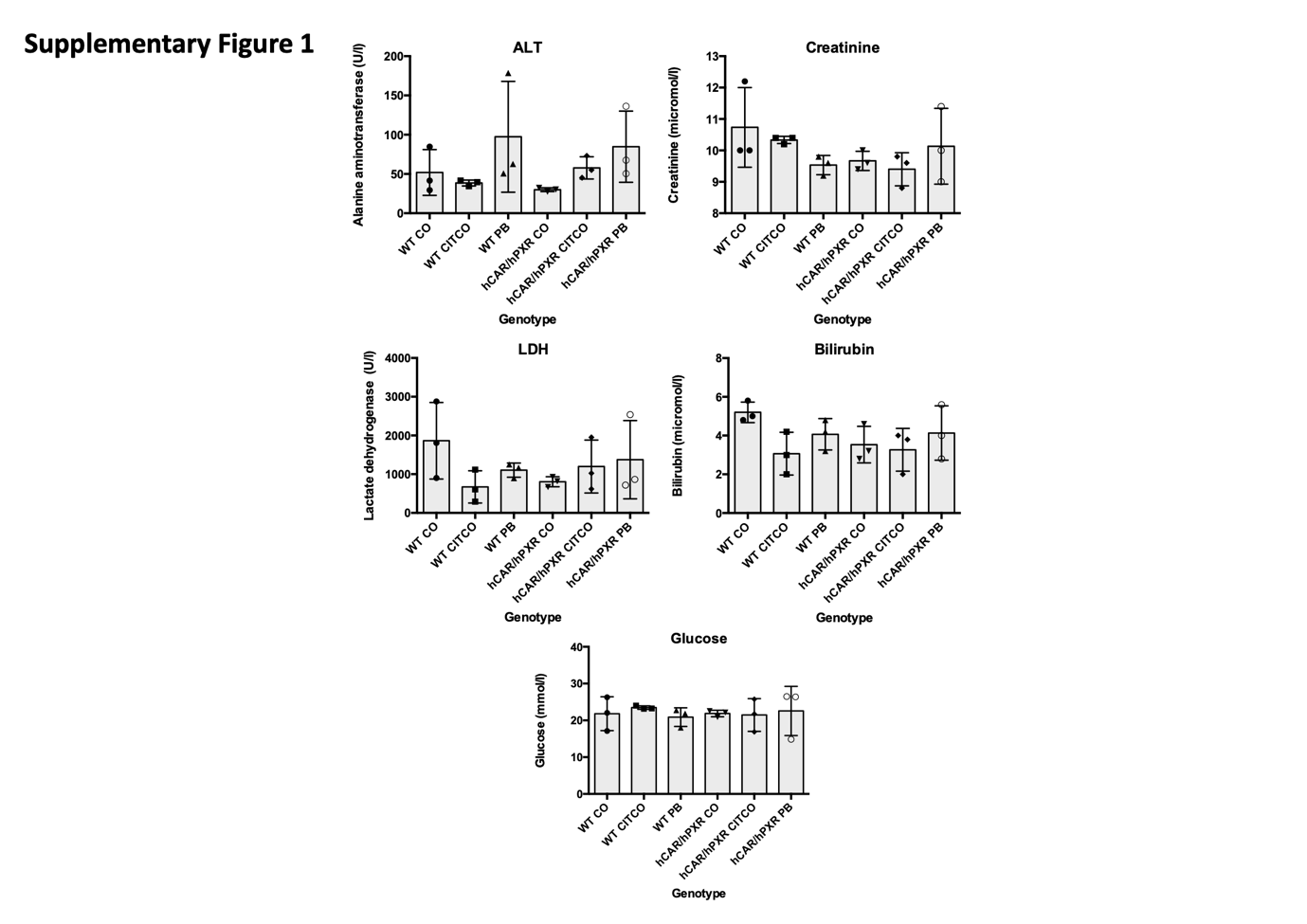


Adult, male wild-type (WT) or mice humanised for CAR and PXR (hCAR/hPXR) (n=3), were treated with corn oil (CO), CITCO (10mg/kg, ip, daily for 4d) or phenobarbital (PB; 80mg/kg, ip daily, 4d) and sacrificed on day 5.

Blood chemistry for alanine aminotransferase (ALT), creatinine, lactate dehydrogenase (LDH), bilirubin and glucose. Symbols for each group indicate data points, error bars mean ± SD, and unpaired t-test indicates no significant difference for CITCO- or PB-treated groups relative to appropriate CO-treated control group.
