# Supplementary material for: The potent human CAR activator CITCO is a non-genotoxic hepatic tumour-promoting agent in humanised constitutive androstane receptor mice but not in wild-type animals
Source: Arch Toxicol. 2025 Mar 5;99(5):2197–210. doi: 10.1007/s00204-025-03982-9 (PMC12085376; doi:10.1007/s00204-025-03982-9)
Supplement: Supplementary file 2 — Supplementary file2 (DOCX 5151 KB) [file 204_2025_3982_MOESM2_ESM.docx]

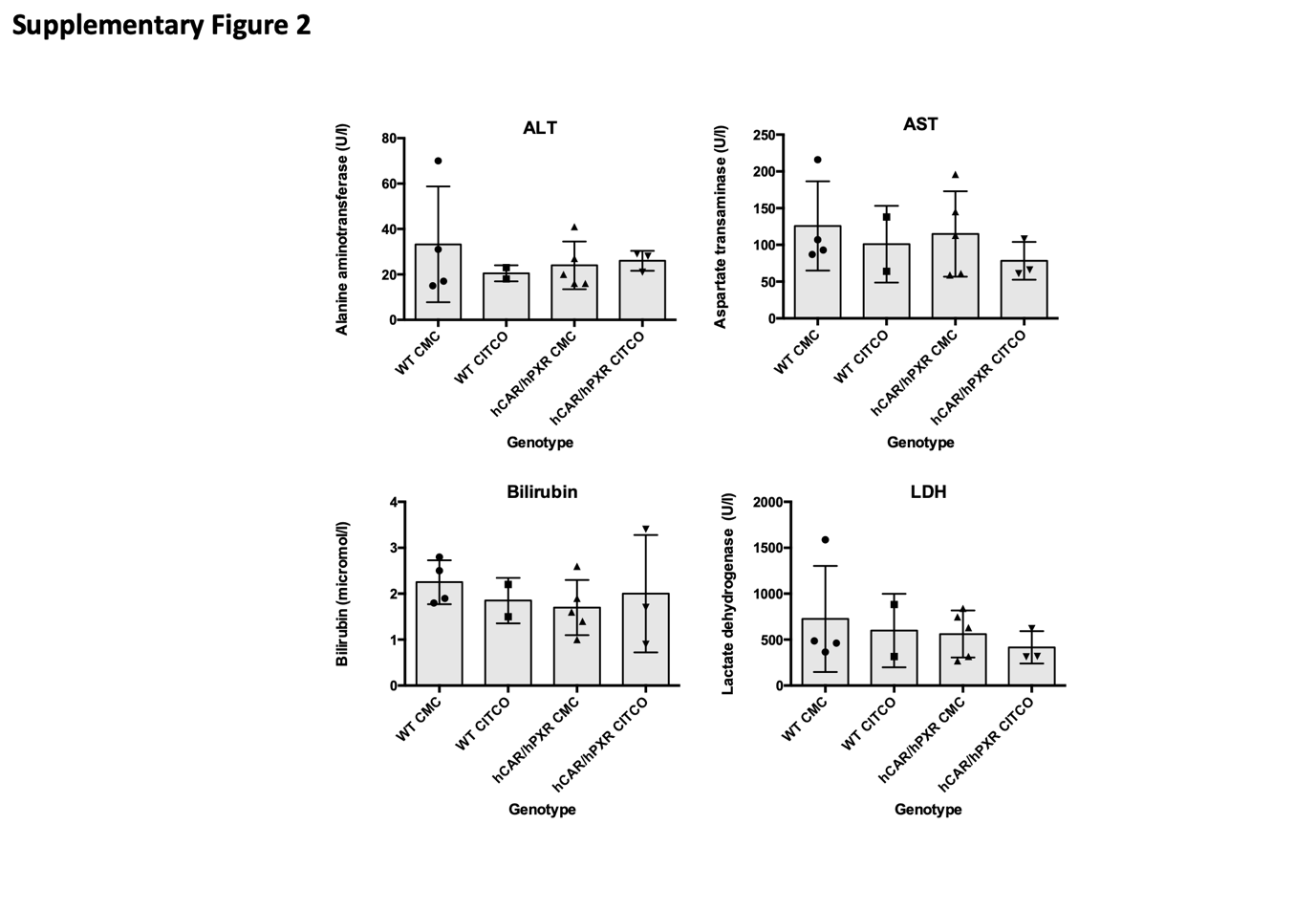


Adult, male wild-type (WT) mice and mice humanised for CAR and PXR (hCAR/hPXR), n=3, were treated with 0.1% (w/v) carboxymethylcellulose (CMC) or CITCO (10mg/kg, ip, daily for 28d) and sacrificed the next day. Blood chemistry for alanine aminotransferase (ALT), aspartate aminotransferase (AST), bilirubin and lactate dehydrogenase (LDH).
