# Supplementary material for: The potent human CAR activator CITCO is a non-genotoxic hepatic tumour-promoting agent in humanised constitutive androstane receptor mice but not in wild-type animals
Source: Arch Toxicol. 2025 Mar 5;99(5):2197–210. doi: 10.1007/s00204-025-03982-9 (PMC12085376; doi:10.1007/s00204-025-03982-9)
Supplement: Supplementary file 3 — Supplementary file3 (DOCX 21 KB) [file 204_2025_3982_MOESM3_ESM.docx]

| **ID** | **Gene** | **Name** | **Abs change** | **Adj p value** |
| --- | --- | --- | --- | --- |
| ENSMUSG00000022809 | **Nr1i2** | nuclear receptor subfamily 1, group I, member 2 | 0.003 | 3.28E-09 |
| ENSMUSG00000005677 | **Nr1i3** | nuclear receptor subfamily 1, group I, member 3 | 0.017 | 2.48E-18 |
| ENSMUSG00000024986 | **Hhex** | hematopoietically expressed homeobox | 0.171 | 4.17E-07 |
| ENSMUSG00000043013 | **Onecut1** | one cut domain, family member 1 | 0.215 | 2.62E-06 |
| ENSMUSG00000020205 | **Phlda1** | pleckstrin homology like domain, family A, member 1 | 0.217 | 8.19E-08 |
| ENSMUSG00000049791 | **Fzd4** | frizzled class receptor 4 | 0.372 | 2.73E-03 |
| ENSMUSG00000047368 | **Abhd17b** | abhydrolase domain containing 17B | 0.380 | 3.06E-03 |
| ENSMUSG00000037025 | **Foxa2** | forkhead box A2 | 0.384 | 3.09E-03 |
| ENSMUSG00000057982 | **Zfp809** | zinc finger protein 809 | 0.403 | 2.73E-03 |
| ENSMUSG00000030004 | **Nat8** | N-acetyltransferase 8 | 0.421 | 1.04E-02 |
| ENSMUSG00000057228 | **Aadat** | aminoadipate aminotransferase | 0.435 | 7.95E-03 |
| ENSMUSG00000018171 | **Vmp1** | vacuole membrane protein 1 | 0.445 | 4.67E-05 |
| ENSMUSG00000002992 | **Apoc2** | apolipoprotein C-II | 0.462 | 2.89E-02 |
| ENSMUSG00000010601 | **Apol7a** | apolipoprotein L 7a | 0.488 | 2.03E-02 |
| ENSMUSG00000010025 | **Aldh3a2** | aldehyde dehydrogenase family 3, subfamily A2 | 0.491 | 1.64E-02 |
| ENSMUSG00000025993 | **Slc40a1** | solute carrier family 40 (iron-regulated transporter), member 1 | 0.491 | 1.98E-02 |
| ENSMUSG00000019947 | **Arid5b** | AT rich interactive domain 5B | 0.496 | 4.93E-02 |
| ENSMUSG00000070780 | **Rbm47** | RNA binding motif protein 47 | 0.503 | 1.05E-03 |
| ENSMUSG00000078673 | **Mup19** | major urinary protein 19 | 0.514 | 4.61E-02 |
| ENSMUSG00000069917 | **Hba-a2** | hemoglobin alpha, adult chain 2 | 0.543 | 2.48E-02 |
| ENSMUSG00000052305 | **Hbb-bs** | hemoglobin, beta adult s chain | 0.546 | 2.48E-02 |
| ENSMUSG00000062181 | **Ces3b** | carboxylesterase 3B | 0.563 | 1.05E-03 |
| ENSMUSG00000029311 | **Hsd17b11** | hydroxysteroid (17-beta) dehydrogenase 11 | 0.563 | 4.02E-02 |
| ENSMUSG00000064294 | **Aox3** | aldehyde oxidase 3 | 0.593 | 4.61E-02 |
| ENSMUSG00000018796 | **Acsl1** | acyl-CoA synthetase long-chain family member 1 | 0.623 | 3.67E-02 |
| ENSMUSG00000033860 | **Fgg** | fibrinogen gamma chain | 1.535 | 1.47E-02 |
| ENSMUSG00000031938 | **4931406C07Rik** | RIKEN cDNA 4931406C07 gene | 1.574 | 2.37E-02 |
| ENSMUSG00000037942 | **Crp** | C-reactive protein, pentraxin-related | 1.676 | 2.48E-02 |
| ENSMUSG00000030339 | **Ltbr** | lymphotoxin B receptor | 1.749 | 3.67E-02 |
| ENSMUSG00000079012 | **Serpina3m** | serine (or cysteine) peptidase inhibitor, clade A, member 3M | 1.780 | 1.26E-04 |
| ENSMUSG00000040017 | **Saa4** | serum amyloid A 4 | 1.890 | 3.71E-02 |
| ENSMUSG00000066441 | **Rdh11** | retinol dehydrogenase 11 | 1.995 | 3.80E-02 |
| ENSMUSG00000055730 | **Ces2a** | carboxylesterase 2A | 2.112 | 1.49E-03 |
| ENSMUSG00000010175 | **Prox1** | prospero homeobox 1 | 2.319 | 1.37E-04 |
| ENSMUSG00000019960 | **Dusp6** | dual specificity phosphatase 6 | 2.445 | 2.37E-02 |
| ENSMUSG00000029630 | **Cyp3a25** | cytochrome P450, family 3, subfamily a, polypeptide 25 | 2.534 | 3.81E-07 |
| ENSMUSG00000026614 | **Slc30a10** | solute carrier family 30, member 10 | 3.042 | 2.97E-02 |
| ENSMUSG00000067279 | **Ppp1r3c** | protein phosphatase 1, regulatory (inhibitor) subunit 3C | 3.233 | 2.37E-02 |
| ENSMUSG00000048489 | **8430408G22Rik** | RIKEN cDNA 8430408G22 gene | 3.837 | 2.48E-02 |
| ENSMUSG00000003053 | **Cyp2c29** | cytochrome P450, family 2, subfamily c, polypeptide 29 | 4.074 | 1.63E-16 |
| ENSMUSG00000056035 | **Cyp3a11** | cytochrome P450, family 3, subfamily a, polypeptide 11 | 4.572 | 1.11E-15 |
| ENSMUSG00000061292 | **Cyp3a59** | cytochrome P450, family 3, subfamily a, polypeptide 59 | 5.643 | 5.12E-03 |
| ENSMUSG00000004038 | **Gstm3** | glutathione S-transferase, mu 3 | 8.252 | 1.68E-10 |
| ENSMUSG00000029380 | **Cxcl1** | chemokine (C-X-C motif) ligand 1 | 9.941 | 4.17E-07 |
| ENSMUSG00000020429 | **Igfbp1** | insulin-like growth factor binding protein 1 | 10.104 | 2.21E-16 |
| ENSMUSG00000030483 | **Cyp2b10** | cytochrome P450, family 2, subfamily b, polypeptide 10 | 57.128 | 1.22E-19 |
| ENSMUSG00000089960 | **Ugt1a1** | UDP glucuronosyltransferase 1 family, polypeptide A1 | 4279242 | 5.27E-04 |

**Supplementary Table 1**

RNAseq analysis carried out on hCAR/hPXR and WT mice treated with CITCO for 4d as described in Materials and Methods. Data shown are gene expression changes up or down more than abs(log2) of 1.5-fold in hCAR/hPXR mice relative to WT, with Benjamini-Hocberg adjusted p values
