# Supplementary material for: The potent human CAR activator CITCO is a non-genotoxic hepatic tumour-promoting agent in humanised constitutive androstane receptor mice but not in wild-type animals
Source: Arch Toxicol. 2025 Mar 5;99(5):2197–210. doi: 10.1007/s00204-025-03982-9 (PMC12085376; doi:10.1007/s00204-025-03982-9)
Supplement: Supplementary file 4 — Supplementary file4 (DOCX 17 KB) [file 204_2025_3982_MOESM4_ESM.docx]

|  | **Pathway name** | **adj.p.val** | **ratio** |
| --- | --- | --- | --- |
| **1** | PXR-mediated direct regulation of xenobiotic metabolizing enzymes / Rodent version | 9.777e-6 | 5/39 |
| **2** | PXR-mediated direct regulation of xenobiotic metabolizing enzymes / Human version | 9.777e-6 | 5/42 |
| **3** | Development_Signaling pathways in embryonic hepatocyte maturation | 1.769e-5 | 5/51 |
| **4** | SREBP1 cross-talk with PXR, CAR and LXR | 3.752e-5 | 4/26 |
| **5** | SREBP1 cross-talk with PXR, CAR and LXR/ Rodent version | 6.258e-5 | 4/31 |
| **6** | CAR-mediated direct regulation of xenobiotic metabolizing enzymes / Human version | 1.276e-4 | 4/40 |
| **7** | CAR-mediated direct regulation of xenobiotic metabolizing enzymes / Rodent version | 1.276e-4 | 4/40 |
| **8** | PXR signaling via cross-talk / Human Version | 6.391e-4 | 3/22 |
| **9** | PXR signaling via cross-talk / Rodent version | 6.391e-4 | 3/22 |
| **10** | Development_Generation of pancreatic beta-cells from embryonic stem cells (late stages) | 6.391e-4 | 3/22 |
| **11** | Fenofibrate in treatment of type 2 diabetes and metabolic syndrome X | 8.630e-4 | 3/25 |
| **12** | CAR signaling via cross-talk / Human Version | 8.927e-4 | 3/26 |
| **13** | CAR signaling via cross-talk / Rodent version | 9.253e-4 | 3/27 |
| **14** | Immune response_IL-6-induced acute-phase response in hepatocytes | 2.063e-3 | 3/36 |
| **15** | Regulation of metabolism_Bile acids regulation of glucose and lipid metabolism via FXR | 2.849e-3 | 3/41 |
| **16** | Retinol metabolism | 1.301e-2 | 3/70 |
| **17** | Development_Generation of pancreatic beta-cells from embryonic stem cells (early stages) | 2.046e-2 | 2/21 |
| **18** | Inhibition of oligodendrocyte precursor cells differentiation by Wnt signaling in multiple sclerosis | 2.704e-2 | 2/25 |
| **19** | Development_Generation of pancreatic beta-cells from induced pluripotent stem cells | 2.704e-2 | 2/27 |
| **20** | Bone metastases in Prostate Cancer | 2.704e-2 | 2/29 |

**Supplementary Table 2**

Table shows the enrichment of Pathways using significant differentially regulated genes. The adjusted p values shows the results of a hypergeometric test. Ratio shows the number of significant differentially regulated genes, over the total number of genes involved in the pathway.
